# Analysis of clinical characteristics and laboratory examination data of 112 cases of Mycoplasma pneumoniae pneumonia in children

**DOI:** 10.1097/MD.0000000000040628

**Published:** 2024-11-22

**Authors:** Pingping Wang, Jin Yao, Yaqiong Li, Xiaorong Huang, Chenhui Deng

**Affiliations:** a Department of Medical Laboratory, Luoyang Maternal and Child Health Hospital, Luoyang City, Henan Province, China; b Department of Infection and Public Health Management, The Second Affiliated Hospital of Henan University of Science and Technology, Luoyang City, Henan Province, China; c Medical Imaging Department, Luoyang Maternal and Child Health Hospital, Luoyang City, Henan Province, China; d Luoyang Research Center for Inheritance and Innovation of Chinese Historical Civilization, Luoyang Institute of Science and Technology School of Marxism (LIT), Luoyang City, Henan Province, China.

**Keywords:** children, clinical characteristics, laboratory examination data, Mycoplasma pneumoniae pneumonia

## Abstract

The aim of this study was to analyze the clinical characteristics of Mycoplasma pneumoniae (MP) infection in children and provide a basis for the diagnosis and treatment of MP and refractory Mycoplasma pneumoniae pneumonia (MPP) in children. A total of 112 children with MPP admitted to Luoyang Maternal and Child Health Hospital between January 31, 2023 and December 31, 2023 were studied, and their clinical characteristics were retrospectively analyzed, including children’s general data, clinical symptoms, imaging changes, bronchoscopy, and laboratory data, including inflammatory factors such as C-reactive protein (CRP), procalcitonin (PCT), interleukin-6 (IL-6), bacterial culture results of bronchoalveolar lavage or sputum, and results of MP culture and detection of MP drug resistance gene loci 23sRNA A2063G and A2064G. Among the 112 children with MPP included in the analysis, 48 were males (42.86%), 64 were females (57.14%), with an average age of 5.74 ± 2.62 years old, 55 cases were older than 6 years old (49.11%), 35 cases were 3 to 6 years old (31.25%), and 22 cases were <3 years old (19.64%), with the older children being the most; 103 cases were fever (91.96%), 94 cases were cough (83.93%), and 46 cases were dyspnea (41.07%). Fiber bronchoscopy showed that 112 cases were mucosal congestion and edema (100.00%), 69 cases were sputum embolism (61.61%), and 19 cases were mucosal erosion (16.96%); the incidence of CRP, PCT, IL-6, and bacterial infection in the MPP group were lower than those in the RMPP group (*P* < .05), and there was no significant difference in the mutation rate of 23sRNA A2063G and A2064G between the 2 groups (*P* > .05); logistic regression analysis showed that CRP, PCT, IL-6, alanine aminotransferase, lactate dehydrogenase, immunoglobulin A, IgG, and immunoglobulin M were the main influencing factors of RMPP (*P* < .05); the length of hospital stay, biochemical treatment of alanine aminotransferase, lactate dehydrogenase, cardiac troponin I, and inflammatory factors of CRP, PCT, and IL-6 in the bronchoalveolar lavage treatment group were lower than those in the general treatment group (*P* < .05). Children with MPP have more fever, cough, and dyspnea as the starting point, and mucosal congestion, edema, sputum embolism are the most common in bronchoscopy. Children with RMPP may be accompanied by decreased immune function and increased inflammation.

## 1. Introduction

Mycoplasma pneumoniae (MP) is a common pathogen that causes respiratory infections. Mycoplasma pneumoniae pneumonia (MPP) accounts for 10% to 40% of community-acquired pneumonia in children under 18 years old.^[1]^ MPP usually has mild symptoms and manifests as a self-limited disease. However, in recent years, an increasing number of severe MPP^[2,3]^ and refractory MPP (RMPP)^[4]^ have emerged in clinical practice, posing great challenges to pediatricians. RMPP is a disease that does not improve after 7 days of treatment with standard macrolide drugs.^[5]^ Macrolides are the preferred drugs for treating childhood MP infections, but with increasing clinical use, drug resistance issues have become more severe, and RMPP cases are also increasing year by year.^[6]^ The most common clinical manifestations of MPP include dry cough and fever, usually accompanied by headache, myalgia, sore throat, as well as laboratory tests (mainly elevated inflammatory markers) and abnormal imaging tests (chest computed tomography shows thickening of the bronchial wall, central lobular nodules, lymph node enlargement or ground glass attenuation, etc).^[7]^ The duration of fever and hospitalization in RMPP is much longer, and the likelihood of developing extrapulmonary complications, including pleural effusion and multiple organ dysfunction, may also lead to more serious long-term sequelae, including obliterative bronchiolitis and bronchiectasis,^[8,9]^ posing a serious threat to the life and health of children.^[10]^

After invading respiratory epithelial cells, MP can stimulate the production of immunoglobulin M (IgM) antibodies in the body, as well as the infiltration of neutrophils and macrophages in local tissues. A large amount of inflammatory cytokines can cause local inflammatory reactions, leading to elevated levels of TNF-α, C-reactive protein (CRP), procalcitonin (PCT), and interleukin-6 (IL-6),^[11,12]^. Long-term inflammatory stimulation may lead to a decrease in immune function and exacerbation of inflammation. When children are infected with MPP, MP can colonize and reproduce in their bodies. The immune system in children can respond, and serum IgM appears first as a specific antibody secreted by B cells. Therefore, early cases of MP infection can detect the presence of IgM antibodies in the serum, which will rise to peak levels after 3 to 4 weeks of continuous infection. The patient’s immune function correspondingly decreases, and serum IgM antibodies will increase as the patient’s condition worsens.^[13,14]^ Immunoglobulin G (IgG) antibodies can neutralize free exotoxins and have a regulatory effect on phagocytic cells. Their low-level expression can affect the function of phagocytes, reduce immune function, and thus increase the risk of pathogen infection.^[15]^ Immunoglobulin A (IgA) is mainly secreted locally by the mucosa. Once IgA antibody deficiency occurs in the body, it is easy for pathogens to invade the respiratory tract and cause infection.^[16]^ Therefore, it is important to seek more effective treatment for children with MPP to prevent their development into RMPP.

After the COVID-19, with the opening of the national epidemic prevention policy at the end of 2022, a strong rebound of pediatric respiratory infectious diseases occurred in many regions of the country, showing a high incidence of RMPP and severe pneumonia. Based on this, this study retrospectively analyzed the clinical data of children with MPP in this region after the epidemic, and analyzed the main influencing factors of MPP developing into RMPP. Clinical studies have shown that bronchoalveolar lavage (BAL) can improve the recovery of lung injury in patients with multipolar consolidation MPP.^[17]^ Therefore, this study further analyzes the effectiveness of BAL in the treatment of RMPP, in order to provide clinical basis for the diagnosis and treatment of MPP and RMPP in children.

## 2. Materials and methods

Retrospective analysis of clinical data of 112 children with MPP admitted to Luoyang Maternal and Child Health Hospital from 31/01/2023 to 31/12/2023, including 48 males (42.86%) and 64 females (57.14%), aged 0 to 15 years, with an average age of 5.74 ± 2.62 years. All enrolled patients underwent MP-DNA testing, biochemical alanine aminotransferase (ALT), lactate dehydrogenase (LDH), cardiac troponin I (CTNI), immunoglobulin IgA, IgG, IgM measurements, inflammatory factors CRP, PCT, and IL-6 measurements, as well as bacterial culture and identification of bronchoalveolar lavage fluid or sputum. The initial admission test results of all subjects, CRP, PCT, and IL-6 test results after 7 days of treatment, and the total length of hospital stay were recorded.

This study was approved by the Medical Ethics Committee of Luoyang Maternal and Child Health Hospital (LWOPN 2024020802.0). As it is an Observational Study, the Medical Ethics Committee has waived patient consent.

### 2.1. Inclusion criteria

All enrolled children with MPP and RMPP were diagnosed and treated in accordance with the diagnostic criteria of the Diagnosis and Treatment Guidelines for Mycoplasma Pneumonia in Children (China 2023 Edition).

It meets the following clinical and imaging manifestations:

Clinical manifestations of MPP:

① It is more common in children aged 5 and above, but it can also occur in children under 5 years old.

② Fever and cough are the main clinical manifestations, which may be accompanied by headache, runny nose, sore throat, ear pain, etc.

③ Fever is mainly moderate to high fever, and those with sustained high fever indicate severe illness.

④ The cough is more severe and may resemble a whooping cough.

⑤ Some children may experience wheezing, which is more common in infants and young children.

⑥ Early signs of the lungs may not be obvious, and as the condition progresses, there may be a decrease in respiratory sounds and dry/wet rales.

Imaging manifestations:

① Early chest X-ray or CT scan of MPP mainly shows thickening and increase of the texture around the bronchial vessels, thickening of the bronchial wall, and may include ground glass shadows, “tree bud signs,” thickening of interlobular septa, and grid shadows.

② The alveoli may have ground glass like shadows, patchy, segmental, and even lobar consolidation, with common atelectasis and possible enlargement of the pulmonary hilum. In severe cases, it may be accompanied by pleural effusion. Unilateral lesions are more common than bilateral lesions.

③ Some MPP may present with localized or diffuse bronchiolitis features.

Combined with any 1 or both of the following, MPP can be diagnosed:

① Single serum MP antibody titer ≥ 1:160 (PA method); During the course of the disease, the titers of double serum MP antibodies increase by 4 times or more.

② MP-DNA or RNA positive.

RMPP refers to patients who have been receiving regular treatment with macrolide antibiotics for 7 days or more, but still experience persistent fever, worsening clinical and pulmonary imaging findings, and extrapulmonary complications.

### 2.2. Exclusion criteria

① Children with congenital immunodeficiency, organ dysfunction, tumors, genetic metabolic diseases, and long-term use of hormones or immunosuppressants.

② Combined with other viral infections such as hepatitis A virus, hepatitis B virus, hepatitis C virus, etc.

③ Incomplete clinical medical records.

### 2.3. Instruments and reagents

CRP detection was performed using Mindray BC-7500 fully automatic blood cell analyzer and supporting reagents, and latex agglutination method; PCT, IL-6, and CTNI were detected using Wantai Wan200 + fully automatic chemiluminescence immunoassay analyzer and matching reagents; The detection of ALT, LDH, immunoglobulin IgA, IgG, and IgM was performed using Hitachi LABOSPECT 008 AS fully automatic biochemical analyzer and matching reagents; Microbial culture and identification were detected using the Merie VITEK2 COMPACT automatic microbial identification analyzer. The nucleic acid detection of MP was carried out using the MP nucleic acid detection kit (PCR fluorescent probe method) produced by Da’an Gene Co., Ltd. of Sun Yat sen University to detect the MP-DNA content in the patient’s throat swab, with a detection result greater than 1 × 10^3^ copies/mL is positive, and the drug-resistant mutation sites of MP in pharyngeal swabs are detected. A positive result at any locus of 23SrRNA 2063 and 2064 indicates a positive resistance gene.

### 2.4. Methods

Laboratory testing: All enrolled patients undergo CRP testing, including immunoglobulin IgA, IgG, IgM, biochemical indicators ALT, LDH, CTNI, inflammatory indicators PCT, IL-6, and MP nucleic acid testing, with strict quality control to ensure accurate results. All selected patients were collected pharyngeal swabs from the posterior pharyngeal wall, tonsil recess, lateral wall, and other areas. Selected patients undergo bacterial culture and identification of bronchoalveolar lavage fluid or cough deep sputum before medication. All patients were collected pharyngeal swabs or bronchoalveolar lavage fluid for qualitative detection of 23sRNA A2063G and A2064G mutation sites resistant to MP macrolide antibiotics.

Laboratory testing: 4 mL of venous blood was drawn from all enrolled patients on an empty stomach in the early morning and placed in EDTA-K2 vacuum anticoagulant tubes and procoagulant tubes, with 1 tube of anticoagulant venous blood used for CRP detection and the other tube of procoagulant venous blood used for immunoglobulin IgA, IgG, IgM, ALT, LDH, CTNI, PCT, IL-6 detection, to ensure completion within 4 hours. At the same time, quality control samples were tested to ensure no loss of control. All enrolled patients were collected throat swabs from the posterior pharyngeal wall, tonsil recess, lateral wall, and other areas for MP nucleic acid testing. Before medication, enrolled patients should take bronchoalveolar lavage fluid or cough deep sputum into sterile containers and immediately send them for testing. They should be inoculated into blood plates and chocolate plates for bacterial culture and identification. All patients were collected pharyngeal swabs or bronchoalveolar lavage fluid for qualitative detection of the 23sRNA A2063G and A2064G mutation sites of MP anti macrolide antibiotic resistance.

Imaging examination: The CT plain scan uses Siemens 64 row spiral CT machine, and the subject is placed in a supine position. Before the scan, the patient is given respiratory training, and the patient’s family can accompany the patient throughout the scan to appropriately soothe their tense emotions and ensure the smooth progress of the examination. CT scan range: from the bottom of the lungs to the tip of the lungs. Scanning parameters: tube voltage 120 KV, tube current 170 mA, scanning pitch 0.875, layer thickness 5 mm, layer pitch 5 mm, matrix 512 × 512, all underwent mediastinal and lung window scans. Lung windows and lung positions can be adjusted according to the patient’s personal conditions.

Treatment plan: The regular treatment group will receive treatment with methylprednisolone [National Medical Standard H20010098, Specification: 0.5 g (calculated as methylprednisolone C_22_H_30_O_5_)]. According to the standard medication of 2 mg/kg, intravenous infusion, once a day, for a total of 3 days of treatment. At the same time, symptomatic treatment methods such as fever reduction, cough relief, and phlegm reduction should be given. In the BAL treatment group, the therapeutic scheme of fiberoptic bronchoscopy was implemented: prepare the electronic bronchus, fast 6 hours before treatment, and perform spray anesthesia in the nasal cavity and pharynx 0.5 hours, 20 minutes, and 10 minutes before surgery respectively. The drug used was lidocaine with a concentration of 2%. During the treatment, the patient was in a supine position, and 1 side of the nasal cavity was connected with an oxygen tube, and a high concentration of oxygen was given. The other side of the nasal cavity was used to place the bronchoscope. After reaching the lesion site, inject physiological saline with a perfusion volume of <5 mL/kg, rinse repeatedly 3 to 5 times, and use negative pressure of 100 mm Hg to inhale physiological saline. Divide the remaining BAL samples into 2 parts, 1 part for pathogen testing and 1 part for drug resistance gene testing. Monitor the child’s heart rate, blood pressure, respiratory, and other physical signs throughout the entire process. A total of 3 days of treatment were given, along with symptomatic treatments such as fever reduction, cough relief, and phlegm reduction. Considering the advantages of bronchoscopy examination, all children included in the analysis in this study met the indications for this examination. The indications for bronchoscopy refer to the Chinese Pediatric Flexible Bronchoscopy Guidelines (2018 Edition).^[18]^ Parents of the affected child sign an informed consent form for bronchoscopy diagnosis and treatment. Both groups of children were retested for serum inflammatory factors CRP, PCT, IL-6, as well as biochemical indicators ALT, LDH, and CTNI after 7 days of treatment.

### 2.5. Statistical methods

Using SPSS 25.0 software for data processing and statistical analysis, describe the number of use cases (n) and percentage (%) of age, gender, and other count data; Measurement data that conform to normal distribution are represented by (*x* ± *s*), while measurement data that do not conform to normal distribution are represented by median and interquartile range M (P25, P75). Group comparison is performed using *t* test, chi square test, and Mann–Whitney *U* test, with *P* < .05 indicating statistical significance.

## 3. Results

General information of all enrolled children was included in the analysis of 112 children with MPP, including 48 males (accounting for 42.86%) and 64 females (accounting for 57.14%), with an average age of 5.74 ± 2.62 years. Among children of different ages, 55 were over 6 years old (accounting for 49.11%), 35 were between 3 and 6 years old (accounting for 31.25%), and 22 were under 3 years old (accounting for 19.64%). The clinical symptoms of all patients were mainly fever, cough, and difficulty breathing. The main CT manifestations of the chest were lung consolidation, accumulation of more than 2 lobes of the lungs, and thickening of the bronchial wall. Under fiberoptic bronchoscopy, the main manifestations were mucosal congestion and edema, as well as sputum embolism. The results are shown in Table 1.

**Table 1:** Clinical symptoms, chest CT and bronchoscopy results of all enrolled children.

	Number of cases	Proportion (%)
Clinical symptoms		
Fever	103	91.96
Cough	94	83.93
Dyspnea	46	41.07
Myalgia	35	31.25
Neck lymph node enlargement	22	19.64
Erythra	9	8.04
Hemolytic anemia	2	1.79
Chest CT examination		
Pulmonary consolidation	65	58.03
Involving ≥ 2 lobes of the lungs	39	34.82
Thickening of bronchial tube wall	23	20.54
Pleural effusion	14	12.50
Fiberoptic bronchoscopy		
Mucosal congestion and edema	112	100.00
Sputum embolism	69	61.61
Mucosal erosion	19	16.96
Plastic bronchus	12	10.71
Mucosal fold	9	8.04
Mucosal erosion and necrosis	3	2.68

Among all enrolled MPP patients, 68 developed RMPP, with an incidence rate of 60.71%. The laboratory test results of MPP group and RMPP group patients showed that the CRP, PCT, IL-6, and incidence of concurrent bacterial infections in MPP group patients were lower than those in RMPP group patients, and the difference was statistically significant. However, there was no statistically significant difference in the mutation rates of 23sRNA A2063G and A2064G in the MP resistance gene locus between the 2 groups. The results are shown in Table 2.

**Table 2:** Comparison of laboratory test results between MPP group and RMPP group children.

Group	MPP group (44 cases)	RMPP group (68 cases)	*F*/*t*/*χ*^2^	*P*
CRP (mg/L)	23.49 ± 6.32	35.28 ± 8.83	‐8.228	<.05
PCT (ng/mL)	0.05 (0.04, 0.05)	0.14 (0.13, 0.17)	482.315	<.05
IL-6 (pg/mL)	35.68 ± 2.75	44.32 ± 3.12	223.757	<.05
IgA (g/L)	0.6 ± 0.11	0.41 ± 0.15	7.643	<.05
IgG (g/L)	5.1 ± 1.42	4.35 ± 1.25	2.937	<.05
IgM (g/L)	2.43 ± 0.61	1.59 ± 0.54	7.566	<.05
ALT (U/L)	36.22 ± 5.47	56.94 ± 4.95	‐20.763	<.05
LDH (U/L)	233.62 ± 19.89	319.16 ± 23.32	‐20.057	<.05
CTNI	0.05 (0.04, 0.05)	0.04 (0.03, 0.06)	‐1.56	>.05
Positive bacterial culture [cases (%)]	11 (25.00)	27 (39.71)	4.00	>.05
Positive drug resistance genes [cases (%)]	24 (54.54)	39 (57.35)	4.00	>.05

ALT = alanine aminotransferase, CRP = C-reactive protein, CTNI = cardiac troponin I, IgA = Immunoglobulin A, IgG = immunoglobulin G, IgM = immunoglobulin M, IL-6 = interleukin (IL)-6, LDH = lactate dehydrogenase, MPP = Mycoplasma pneumoniae pneumonia, PCT = procalcitonin, RMPP = refractory Mycoplasma pneumoniae pneumonia.

Pearson correlation was used to analyze the correlation between laboratory indicators and the occurrence of RMPP. The results showed that CRP, PCT, IL-6, ALT, LDH, IgA, IgG, and IgM were all related to the occurrence of RMPP, as shown in Table 3. Further logistic regression analysis was used to identify the main factors influencing the development of MPP into RMPP. The results showed that the main influencing factors were CRP, PCT, IL-6, ALT, LDH, IgA, IgG, and IgM. The results are shown in Table 4.

**Table 3:** Pearson correlation analysis of the correlation between different indicators and the occurrence of RMPP.

	CRP	PCT	IL-6	ALT	LDH	IgA	CTNI	IgG	IgM
Correlation coefficient	0.590	0.902	0.819	0.893	0.886	‐0.567	0.127	‐0.270	‐0.585
*P*	<.01	<.01	<.01	<.01	<.01	<.01	.183	.004	<.01

ALT = alanine aminotransferase, CRP = C-reactive protein, CTNI = cardiac troponin I, IgA = immunoglobulin A, IgG = immunoglobulin G, IgM = immunoglobulin M, IL-6 = interleukin (IL)-6, LDH = lactate dehydrogenase, PCT = procalcitonin, RMPP = refractory Mycoplasma pneumoniae pneumonia.

**Table 4:** Binary logistic regression analysis of MPP developing into RMPP.

Independent variables	β value	SE	Wald	OR	*P*
CRP	0.191	0.037	26.27	1.21	<.05
PCT	0.435	0.193	5.062	1.545	<.05
IL-6	0.949	0.201	22.242	2.582	<.05
ALT	0.89	0.293	9.196	2.434	<.05
LDH	0.235	0.093	6.337	1.265	<.05
CTNI	‐0.259	0.553	0.220	0.771	>.05
IgA	‐10.514	2.072	25.743	0	<.05
IgG	‐0.426	0.155	7.537	0.653	<.05
IgM	‐2.692	0.541	24.778	0.068	<.05

ALT = alanine aminotransferase, BAL = bronchoalveolar lavage, CRP = C-reactive protein, CTNI = cardiac troponin I, IgA = immunoglobulin A, IgG = immunoglobulin G, IgM = immunoglobulin M, IL-6 = interleukin (IL)-6, LDH = lactate dehydrogenase, MPP = Mycoplasma pneumoniae pneumonia, PCT = procalcitonin, RMPP = refractory Mycoplasma pneumoniae pneumonia.

Among the 68 children in the RMPP group, 37 received BAL treatment and 31 received regular treatment. According to the different treatments, the RMPP group children were further divided into regular treatment group children and BAL treatment group children. After corresponding treatment, the hospitalization days, biochemical indicators such as ALT and LDH, as well as inflammatory factors CRP, PCT, and IL-6 in the ordinary treatment group were on average higher than those in the BAL treatment group. The results are shown in Table 5.

**Table 5:** Comparison of main laboratory test results between children in the regular treatment group and children in the BAL treatment group.

Group	Hospitalization time (Day)	ALT (IU/L)	LDH (IU/L)	CTNI (IU/L)	CRP (mg/L)	PCT (ng/mL)	IL-6 (pg/mL)
Ordinary treatment group	24.07 ± 4.38	48.29 ± 5.64	292.58 ± 17.11	0.03 (0.01, 0.04)	24.27 ± 7.8	0.11 ± 0.03	36.68 ± 3.17
BAL treatment group	18.96 ± 4.70	39.46 ± 9.58	268.81 ± 20.35	0.02 (0.01, 0.03)	12.64 ± 3.01	0.04 ± 0.02	28.92 ± 5.1
*t*/*F*	‐4.636	4.518	5.234	3.742	8.348	9.761	7.367
*P*	<.05	<.05	<.05	>.05	<.05	<.05	<.05

ALT = alanine aminotransferase, BAL = bronchoalveolar lavage, CRP = C-reactive protein, CTNI = cardiac troponin I, IL-6 = interleukin (IL)-6, LDH = lactate dehydrogenase, PCT = procalcitonin.

## 4. Discussion

MP has occasional small-scale outbreaks in the population, mainly affecting school-age children. The main site of infection is the respiratory system, with common clinical problems such as lung consolidation, pleural effusion, necrotizing pneumonia, and respiratory failure. This study analyzed the general data of enrolled children and found that the outbreak of MPP in children after the epidemic was mainly in older children, with fever, cough, breathing difficulties as the main clinical manifestations, which was consistent with reports from other regions,^[19–21]^ and the incidence of RMPP was still relatively high. Due to resistance to macrolide antibiotics and other “difficult to treat” reasons, RMPP fails to specifically inhibit inflammatory reactions in the early stages, which can easily progress to severe MPP. Lung effusion and/or necrosis, or extrapulmonary complications may occur, and in severe cases, sequelae may occur, increasing treatment cycle, difficulty, and cost. Studies have shown that patients with RMPP can be treated with tetracycline or fluoroquinolone drugs.^[22]^ When patients have no response to fluoroquinolone drugs, tigecycline can also be added.^[23]^ However, this multidrug therapy may disrupt the balance of gut microbiota^[24]^ and increase the risk of adverse drug reactions.^[25]^ Especially, these new drugs may trace back to lifelong sequelae in children, including tetracycline teeth, cartilage dysplasia, and so on. Therefore, it is suggested that clinical physicians should consider both the condition and adverse reactions when using medication in clinical practice.

The results of this study found that the levels of inflammatory factors CRP, PCT, and IL-6 in the RMPP group were higher than those in the MPP group, and the levels of major immunoglobulins were lower than those in the MPP group. Research has shown that as the severity of MPP increases, the levels of immunoglobulin and inflammatory factors in children with MPP will change accordingly. This study further supplements the changes in this indicator when MPP progresses to RMPP. The results of this study found that the RMPP group had higher levels of inflammatory factors and lower levels of immunoglobulin. Children in the RMPP group had higher levels of ALT and LDH than those in the MPP group, which is consistent with the conclusions of other studies.^[26]^ The mechanism of MPP extrapulmonary injury is often believed to share antigens with MP and extrapulmonary organs. After MP invades the human body, it produces self-antigens, triggers immune reactions, forms immune complexes, and causes liver and myocardial damage.^[27]^ Lactate dehydrogenase is a glycolytic enzyme present in the myocardium, liver, kidneys, skeletal muscle, and lung tissues. In children with RMPP, continuous stimulation of MP can lead to excessive defense responses in the body,^[28,29]^ resulting in lung tissue damage and the release of lactate dehydrogenase in damaged cells. The results of this study indicate that inflammatory factors CRP, PCT, IL-6, ALT, LDH, and immunoglobulin IgA, IgG, and IgM are closely related to RMPP, affecting the progression of MPP children to RMPP. This suggests that children with RMPP may experience more severe inflammatory reactions, immune dysfunction, and more severe liver and myocardial damage.

The results of this study found that the levels of ALT and LDH in children in the RMPP group were higher than those in the MPP group, which was consistent with the conclusions of other studies.^[26]^ The mechanism of extrapulmonary injury in MPP is generally believed to have a common antigen with MP and extrapulmonary organs. After MP invades the body, it produces its own antigen, triggers an immune response, and forms an immune complex, leading to liver and myocardial damage.^[27]^ Lactate dehydrogenase is a glycolytic enzyme that exists in the myocardium, liver, kidneys, skeletal muscle, and lung tissues. In children with RMPP, the sustained stimulation of MP causes excessive defense response in the body,^[28,29]^ leading to lung tissue damage and the release of lactate dehydrogenase in damaged cells. The results of this study suggest that children with RMPP may have more severe inflammatory reactions, immune dysfunction, and more severe liver and myocardial damage.

Based on the fact that MP only has 1 rRNA operon, it is generally believed that the molecular mechanism of MP’s resistance to macrolide drugs is due to mutations at sites 2063 and 2064 of the central ring of 23S rRNA region V, leading to target site methylation and drug inactivation.^[30,31]^ However, the results of this study found that there was no significant difference in the mutation rates of MP resistance genes at sites 2063 and 2064 between the RMPP group and the MPP group, indicating that MPP may be a self-limited disease. The 2 mutations in the resistance gene locus are not the only possible resistance mechanism in RMPP, which may involve autoimmune reactions. The immune response produced by organisms may also play an important role.^[32]^ The immune dysfunction of pathogens after reactive infection with MP leads to a series of inflammatory reactions, leading to worsening of lung injury and disease, suggesting that the immune response of pathogens after MP infection may be an important factor in the development of RMPP.

At present, there is controversy over the use of anti-infective immune regulation and other related treatments for MPP patients with antibiotic resistance in clinical practice.^[33]^ This study compared and analyzed different treatment methods in RMPP patients, and found that BAL showed good therapeutic effects in shortening hospitalization time, reducing inflammatory cytokine levels, and promoting biochemical indicators such as ALT and LDH. It suggests that clinical physicians may achieve more satisfactory therapeutic effects by combining anti-infective immunomodulatory therapy with BAL treatment.

In summary, the progression of MPP to RMPP may be the result of a combination of immune dysfunction and inflammatory response in the body, and BAL has a good therapeutic effect in the treatment of RMPP. There are still shortcomings in this study. The impact of drug-resistant gene mutations on RMPP needs to be further tested at multiple loci, and further analysis with large samples and multiple centers is needed in the future.

## Acknowledgments

Thanks to Dr Deng Chenhui of Luoyang Institute of Science and Technology for his support in the field of statistics for this paper. Meanwhile we would like to thank our funders, esp Luoyang Maternal and Child Health Hospital, for their kind and generous financial support towards achieving these study objectives.

## Author contributions

**Data curation:** Chenhui Deng.

**Formal analysis:** Chenhui Deng.

**Methodology:** Jin Yao.

**Project administration:** Xiaorong Huang.

**Supervision:** Xiaorong Huang.

**Writing – original draft:** Pingping Wang.

**Writing – review & editing:** Yaqiong Li.
